# Metabolomics and Transcriptomics Analyses Reveals the Molecular Regulatory Mechanisms of Walnut (*Juglans regia* L.) Embryos in Response to Shade Treatment

**DOI:** 10.3390/ijms241310871

**Published:** 2023-06-29

**Authors:** Manman Liang, Qinglong Dong, Xuemei Zhang, Yang Liu, Han Li, Suping Guo, Haoan Luan, Peng Jia, Minsheng Yang, Guohui Qi

**Affiliations:** College of Forestry, Hebei Agricultural University, Baoding 071001, China; liangmm2023@163.com (M.L.); dong19850412@163.com (Q.D.); zhangxuemei888@163.com (X.Z.); wolongchina@163.com (Y.L.); 13733221922@163.com (H.L.); lbg888@163.com (S.G.); luanhaoan@163.com (H.L.); jiapeng@hebau.edu.cn (P.J.)

**Keywords:** shading, metabolomics, transcriptomics, walnut embryo, network, lipid, protein

## Abstract

The walnut is an important nut that has numerous uses worldwide. However, due to dwarf and close plantation methods as well as continuous cloudy or rainy days that occur during periods of walnut oil accumulation, the walnut fruit exhibits varying degrees of stress under low-light conditions. However, the effects of shade on metabolites and genes in walnut embryos remain unclear in the literature. The purpose of this study is to investigate the lipid biosynthesis process that occurs in walnut embryos under shade treatment via the use of metabolomics and transcriptomics analyses. The results indicate that the oil content decreases significantly under shaded conditions, while the protein content increases significantly. The expression levels of fatty acid desaturase 2 (*FAD2*) and stearoyl-ACP-desaturase (*SAD*) involved in the lipid biosynthesis mechanism were significantly reduced in the shaded group, which resulted in reductions in oleic (C18:1), linoleic (C18:2), and α-linolenic (C18:3) acids. The reduced oil content was consistent with the downregulation of genes associated with the lipid biosynthesis mechanism. In the amino acid biosynthesis process, the upregulated cysteine synthase (*cscK*) and anthranilate synthase beta subunit 2 (*trpG*) genes promoted the accumulation of L-aspartic acid and L-citrulline. The increase in protein content was consistent with the upregulation of genes related to amino acid biosynthesis. Thus, our study provides new insights into the regulatory mechanisms of shade underlying overall walnut fruit quality.

## 1. Introduction

The walnut (*Juglans regia* L.) is an important and widely cultivated woody oilseed tree species in the world. Walnut oil has multiple economic and health benefits [1,2,3,4]. Walnut embryos are rich in nutritional value and bioactive ingredients. Their consumption is beneficial to the brain and intelligence, lowers blood lipid levels, and decreases oxidative stress levels and the risk of cancer [5,6]. Triacylglycerols (TAGs) are the primary type of oil found in plant seeds [7]. The synthesis of plant oil mainly involves two steps: first, the synthesis of fatty acids is conducted in plastids; then, the synthesis of TAGs is performed in the endoplasmic reticulum [8,9]. The lipid synthesis pathway in walnut embryos has been extensively studied in the literature [9,10,11,12]. In recent years, researchers have published studies exploring the walnut genome, a genome that is assembled at the chromosome level [13]. Due to its high efficiency, precision, and dependability factors, transcriptome analysis has been widely used in the study of plants [14,15]. However, due to the complexity of plant biological processes, transcriptome data cannot accurately describe the macro events occurring in biological systems. In recent years, the combination of metabolomics and transcriptome analyses has been widely used in research conducted in the field to study plants, which can more systematically and comprehensively explore the mechanism of metabolite synthesis [16,17,18].

However, the synthesis and accumulation of walnut oil are processes controlled by multiple environmental factors present during the fruit-development stage, such as light intensity, water management, and fertility [19,20,21]. Environmental factors affect plant growth and development processes [22,23]. Light is an indispensable factor for plant growth and development and represents the basis for plant photosynthesis [24]. Insufficient or excessive light-intensity levels affect the processes of plant growth and development as well as nutrient accumulation [25,26]. Additionally, light-intensity levels affect leaves’ water-use efficiency and gas exchange and ultimately affect plant morphogenesis and metabolic processes [27,28]. Fruit quality is related to not only plant varieties but also the cultivation and management of fruits as well as the external environment.

The nutrition and quality outcomes of fruits are influenced by environmental factors and canopy microclimate [29,30]. Reasonable tree shapes and leaf canopy structures can increase the effective radiation area and improve the microenvironment, which is of great significance in producing high-quality and high-yield fruits. Plants are often shaded to varying degrees during their growth stage [31]. Shading stress affects the physiology and molecular mechanisms of plants [32,33]. In their study, Liang et al. [19] reported that shade conditions reduced the overall oil content of walnut fruits. These studies showed that appropriate light conditions were crucial for the growth and development of these fruits. These studies mainly concentrated on the effects of shade on the changes occurring in the primary nutrients present in walnut fruits. However, in recent years walnuts planted in the northeastern regions of China coincided with rainy weather during periods of oil accumulation. Continuous cloudy or rainy days seriously affected the commodity value of walnut fruits. On the other hand, in order to produce a high fruit yield, the planting density of walnut trees was constantly expanded, resulting in different degrees of shade that adversely affected the normal growth and development of the walnut fruits.

Moreover, the role of shade treatment in the genes and metabolites of walnuts has yet to be reported in the literature, suggesting the necessity for further studies regarding transcriptomics and metabolomics analyses. Shade experiments were conducted during periods of walnut oil accumulation ranging from 50 to 120 days after pollination (DAP). The integrative omics perspective is anticipated to successfully explain the changes occurring in the molecular mechanisms of walnut lipid biosynthesis under shade conditions, and we also explore the key genes and metabolites regulating lipid synthesis under shade conditions. Our research provides a basis for understanding the gene and metabolite responses to low-light stress conditions during the walnut fruit-development process.

## 2. Results

### 2.1. Effects of Shade Treatment on Environmental and Photosynthetic Parameters

**Figure 1:**
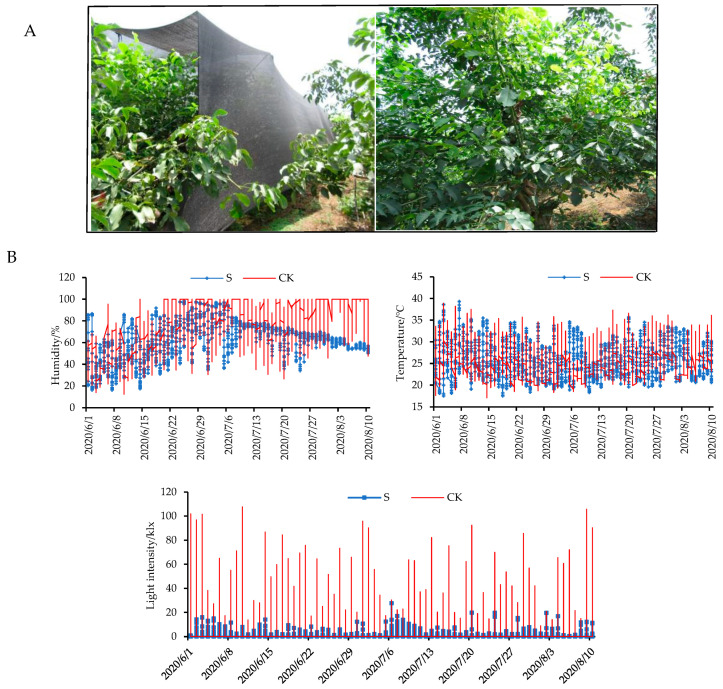
(**A**) Shade setup conditions for walnut trees. (**B**) The effects of shade on environmental parameters (humidity, temperature, and light-intensity levels) in the control (naturally growth) and shaded (walnut trees with 80–90% shade treatment) groups. CK: control check; S: shade treatment.

To investigate the effects of shade on walnut fruit development, shade experiments were conducted in our study. As shown in Figure 1A, walnut trees were exposed to shade in 50–120 DAP (from the early to mature stages of oil accumulation). In order to understand the growth conditions of walnut fruits under natural and shaded conditions, we measured the humidity, temperature, and light-intensity levels (Figure 1B, Table S1). The results indicate that the light-intensity levels to which walnut trees were exposed were significantly reduced by 80–90% in the shaded group compared to the control group.

**Figure 2:**
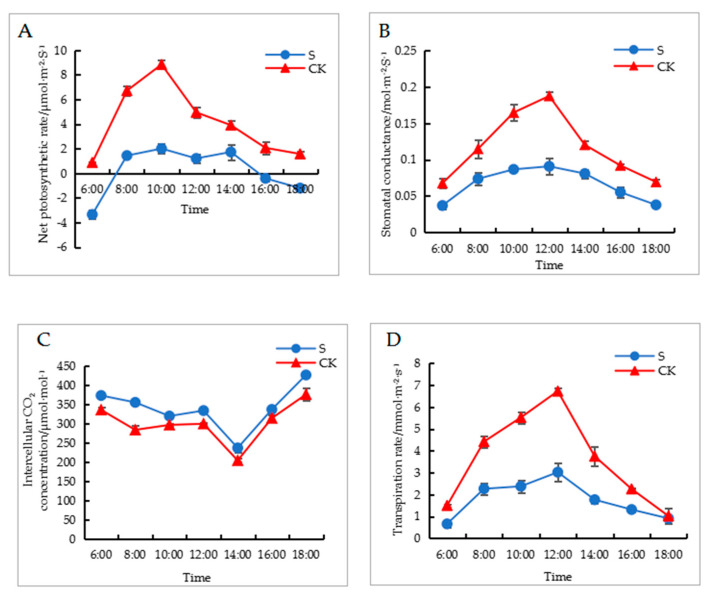
Effects of shade on photosynthetic parameters of walnut leaves. (**A**) is net photosynthetic rate, (**B**) is stomatal conductance, (**C**) is intercellular CO_2_ concentration, and (**D**) is transpiration rate. CK: control check; S: shade treatment.

Shade treatment presented a significant impact on the photosynthetic parameters of walnut leaves. In Figure 2A, it can be observed that the net photosynthetic rate of walnut leaves exhibited an increasing and then decreasing trend. The net photosynthetic rate of walnut leaves in the shaded group was significantly lower than that in the control group. At 10:00, the photosynthetic rates of the control and shaded groups were 8.83 and 2 μmol·m^−2^·s^−1^, respectively. As can be observed in Figure 2B, the stomatal conductance of walnut leaves presents an increasing and then decreasing trend. The stomatal conductance of walnut leaves in the shaded group was significantly lower than that in the control group. As can be observed in Figure 2C, the intercellular CO_2_ concentration level of walnut leaves shows a decreasing and then increasing trend, and the intercellular CO_2_ concentration level for the control group is significantly lower than that of the shaded group. As can be observed in Figure 2D, the transpiration rate of walnut leaves shows a trend of increasing and then decreasing, and the transpiration rate of the control group is significantly higher than that of the shaded group.

### 2.2. Analysis of Differentially Expressed Genes (DEGs) in Response to Shade Treatment

To investigate the effect of shade on walnut fruit development at the transcriptional levels, we utilized RNA-Seq technology to analyze gene activities occurring in walnut embryos derived from both the control and shaded groups. The natural-growth walnut embryos were collected at 60, 90, and 120 DAP, designated as CK1, CK2, and CK3, respectively. The shaded-condition walnut embryos were collected at 60, 90, and 120 DAP, designated as S1, S2, and S3, respectively. Eighteen cDNA libraries were sequenced, and 890, 878, 450 raw reads were generated. Following the data-filtering stage, 815, 978, 812 clean reads were produced, and the Q20 and Q30 values exceeded 99.98% and 97.94%, respectively (Table S2). The proportion of clean reads mapped to the walnut reference genome ranged from 94.25% to 97.22% (Table S3). All these data demonstrate adequate sequencing quality for further analysis.

**Figure 3:**
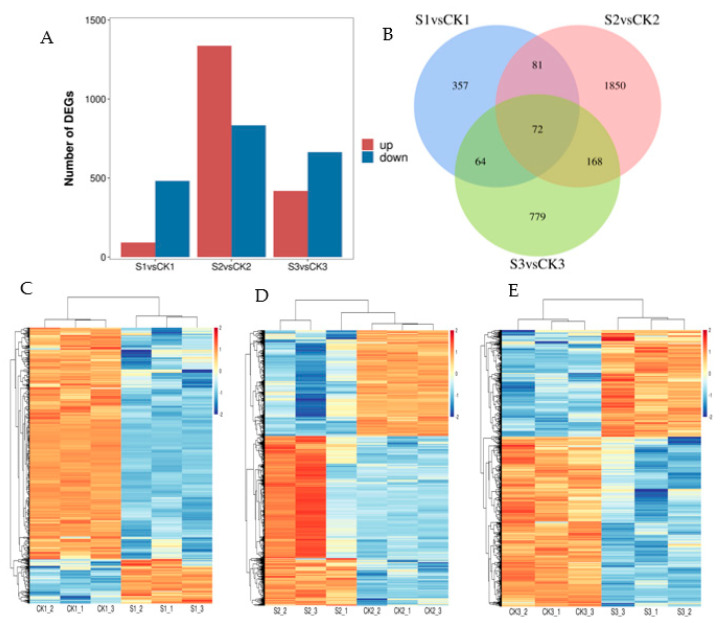
Analysis of differences in transcriptomic data between shaded and control groups. (**A**) Number of DEGs in shaded and control groups. (**B**) Venn diagram of DEGs in shaded and control groups. (**C**) Cluster heat map of DEGs between CK1 and S1 comparison groups. (**D**) Cluster heat map of DEGs between CK2 and S2 comparison groups. (**E**) Cluster heat map of DEGs between CK3 and S3 comparison groups. The red and blue blocks indicate upregulated and downregulated genes, respectively. CK1: walnut embryo showing natural growth at 60 DAP; S1: shaded walnut embryo at 60 DAP; CK2: walnut embryo grown naturally at 90 DAP; S2: shaded walnut embryo at 90 DAP; CK3: walnut embryo exhibiting natural growth at 120 DAP; S3: shaded walnut embryo at 120 DAP. The 60, 90, and 120 DAP indicate time points at 10, 40, and 70 d, respectively, throughout shading period.

The results of the statistical analysis of DEGs between shaded and control groups are presented in Figure 3A. Among the 574 DEGs between S1 and CK1, 92 were upregulated and 482 were downregulated. Among the 2171 DEGs between S2 and CK2, 1337 were upregulated and 834 were downregulated. A total of 1083 DEGs between S3 and CK3 were evident, of which 419 were upregulated and 664 were downregulated. The DEG overlap that was exhibited between the shaded and control groups is presented in Figure 3B. The total number of shared DEGs in all groups was 72. The cluster analysis conducted on DEGs between the shaded and control groups are presented in Figure 3C–E. The transcriptomic analysis we conducted indicated that shade treatment resulted in a high number of gene differential expressions during the walnut fruit development stage.

### 2.3. Analysis of Different Accumulated Metabolites (DAMs) in Response to Shade Treatment

During the walnut fruit-development stage, walnut embryos grown naturally and under shade conditions were analyzed using the LC-MS technique. The total ion chromatogram (TIC) value indicates a high overlap of QC samples and the stable instrument (Figure S1).

**Figure 4:**
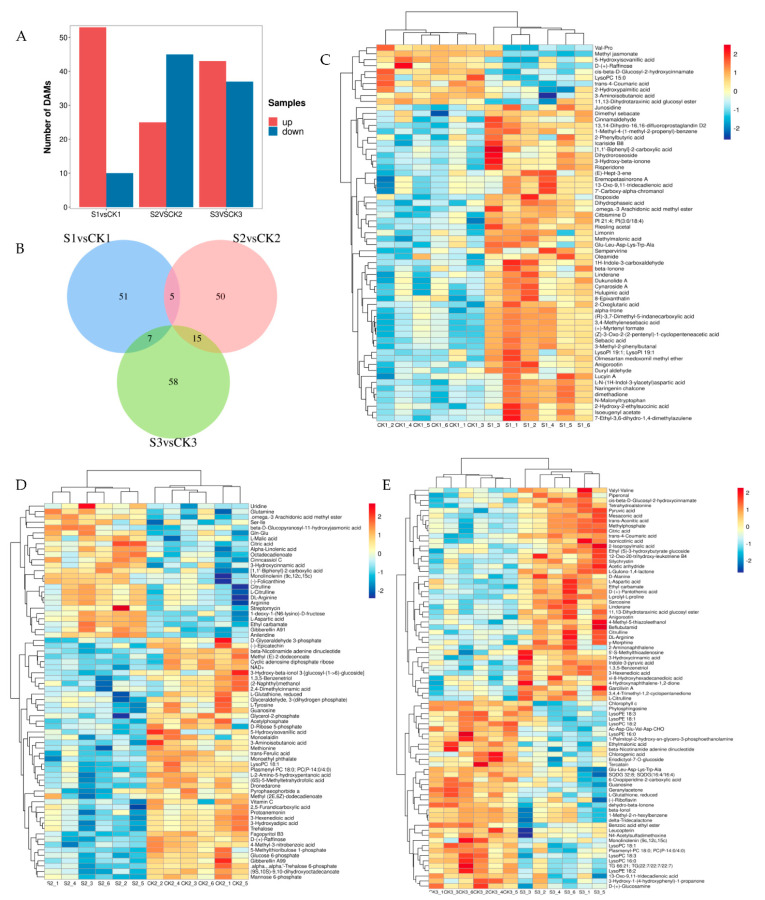
Analysis of differences in metabolomics data between shaded and control groups. (**A**) Number of DAMs in shaded and control groups. (**B**) Venn diagram of DAMs in shaded and control groups. (**C**) Cluster heat map of DAMs between CK1 and S1 comparison groups. (**D**) Cluster heat map of DAMs between CK2 and S2 comparison groups. (**E**) Cluster heat map of DAMs between CK3 and S3 comparison groups. The red and blue blocks indicate upregulated and downregulated metabolites, respectively.

The DAMs between the shaded and control groups are presented in Figure 4A. A total of 63 DAMs between S1 and CK1 comparison groups can be observed, of which 53 were upregulated and 10 were downregulated. Among the 70 DAMs between S2 and CK2, 25 metabolites were upregulated and 45 were downregulated. A total of 79 DAMs between S3 and CK3 were evident, of which 42 were upregulated and 37 were downregulated. Under prolonged treatment conditions, the number of DAMs gradually increased. The DAM overlap behavior between the shaded and control groups is presented in Figure 4B. There were 51, 50, and 58 unique metabolites evident in the S1vsCK1, S2vsCK2, and S3vsCK3 groups, respectively, which indicates that shade treatment affects different developing stages. The cluster analysis conducted on DAMs between the shaded and control groups is presented in Figure 4C–E. The results show that the six replicates of the shaded and control groups are clustered together and that metabolites detected under shade treatment change significantly. Shade treatment primarily influenced the levels of amino and fatty acids in the walnut embryos.

### 2.4. Principal Component Analysis (PCA)

**Figure 5:**
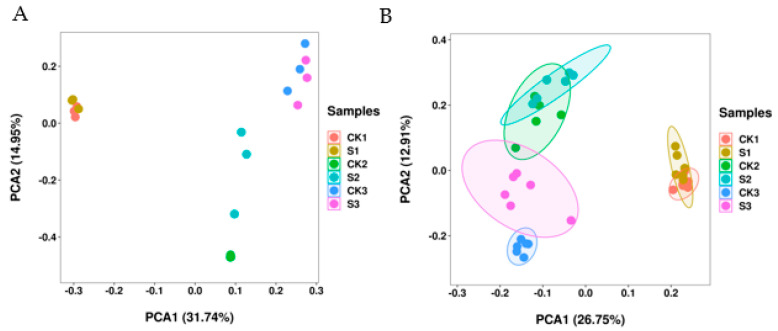
Principal component analyses (PCAs) of walnut fruit under the shade condition. (**A**) PCA score of transcriptomic data between control and shaded groups. (**B**) PCA score of metabolomic data between the control and shaded groups.

Walnut embryos collected under natural-growth and shade conditions were analyzed via RNA-seq and LC-MS methods. RNA-seq FPKM was used to perform a PCA analysis of 18 samples (Figure 5A). PC1 and PC2 explained the 31.74% and 14.95% variations, respectively. Metabolite peak area was used to perform a PCA analysis of 36 samples (Figure 5B). PC1 and PC2 explained the 26.75% and 12.91% variations, respectively. PCA revealed minor differences in the walnut embryos between the control and shaded groups. However, some overlap values in the samples and high intrasample variations in the same group were observed. Walnut embryos in the early stage of fruit development (S1 and CK1) were clearly distinguishable from walnut embryos in middle and mature stages of fruit development (S2, CK2, S3, and CK3) in both PC1 and PC2. The data suggest the evidence of significant differences in the developmental periods as well as specific differences between the shaded and control groups.

### 2.5. Kyoto Encyclopedia of Genes and Genomes (KEGG) Enrichment Analysis of Candidate Pathways in Response to Shade Treatment

**Figure 6:**
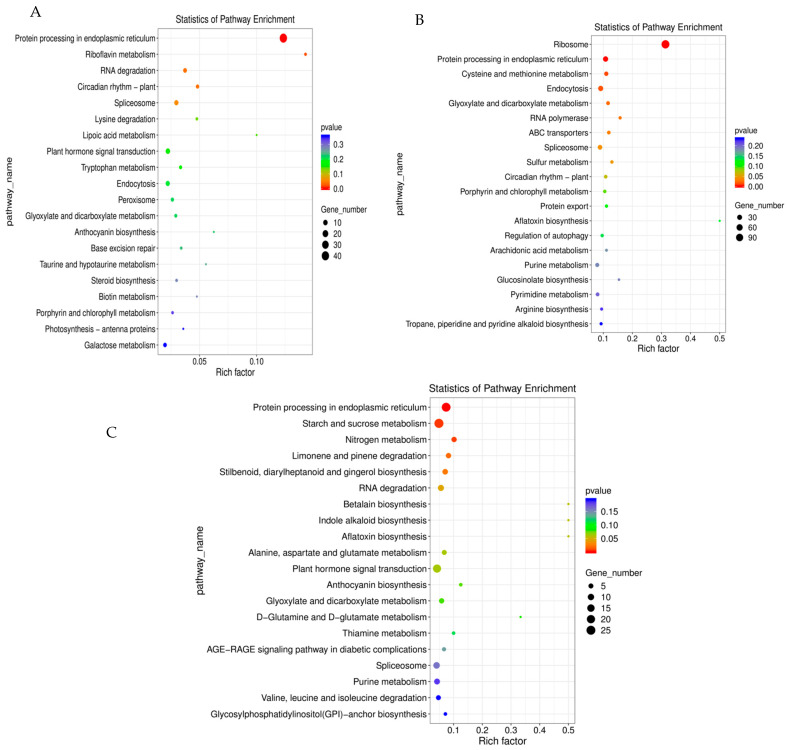
KEGG enrichment analysis of DEGs between shaded and control groups: (**A**) CK1vsS1, (**B**) CK2vsS2, and (**C**) CK3vsS3.

The KEGG enrichment analysis of DEGs between the control and shaded groups is presented in Figure 6. The DEGs of the S1vsCK1 comparison group were mainly enriched in protein-processing activity in the endoplasmic reticulum (ko04141), riboflavin metabolism (ko00740), and RNA degradation (ko03018) (Figure 6A). The DEGs of the S2vsCK2 comparison group were mainly enriched in ribosome (ko03010), protein processing in the endoplasmic reticulum, and cysteine and methionine metabolism (ko00270) (Figure 6B). The DEGs of the S3vsCK3 comparison group were mainly enriched in protein processing in the endoplasmic reticulum, starch and sucrose metabolism (ko00500), and nitrogen metabolism (ko00910) (Figure 6C). After receiving shade treatment, all comparison groups were mainly enriched in protein-processing activity in the endoplasmic reticulum pathway, which indicated that shade mainly affected the protein synthesis activity and contributed to the secondary metabolism of fruit development in response to low-light stress. These results obtained using the KEGG enrichment analysis provide important information for further investigations in the research of the specific pathways involved in walnut fruit development under shade-treatment conditions.

### 2.6. Differences in Metabolic Pathways under Varying Shade-Treatment Durations

In order to further elucidate the effect of shade treatment on the fruit-development process, we studied the key genes and metabolites involved in lipid biosynthesis (ko00061, ko01040, ko00564, and ko00561), plant hormone signal transduction (ko04075), starch and sucrose metabolism (ko00500), glycolysis/gluconeogenesis (ko00010), glutathione metabolism (ko00480), and the biosynthesis of amino acids (ko01230).

#### 2.6.1. Lipid Biosynthesis in Response to Shade Treatment

**Figure 7:**
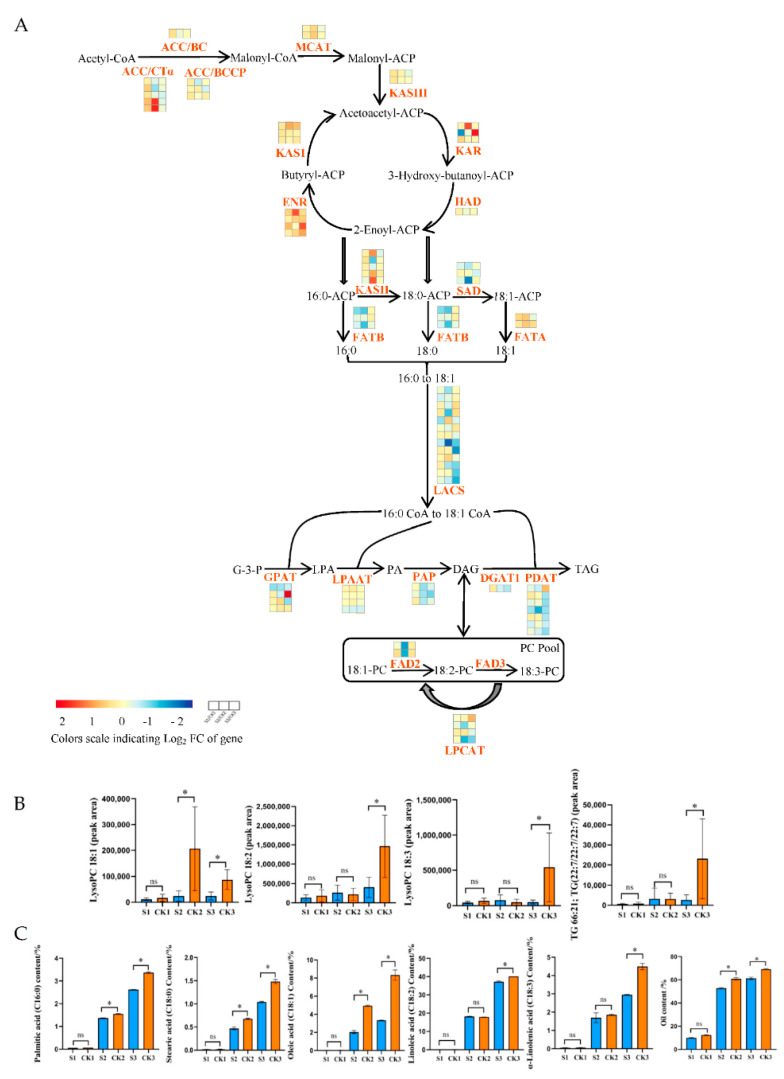

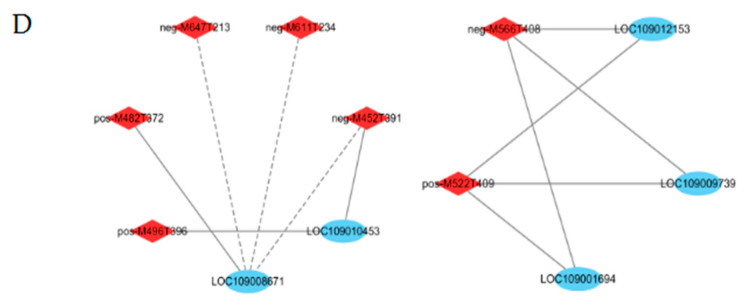
Effects of shade on lipid biosynthesis. (**A**) Differences in gene expression values during three stages of fruit development in fruits produced by shaded- and control-group walnut trees. The three blocks in each horizontal row show log2 fold changes in genes between CK1 and S1, CK2 and S2, and CK3 and S3. The log2 fold change in FPKM values was calculated under shade conditions relative to natural conditions. (**B**) Changes in the peak areas of metabolites under shade treatment. (**C**) Changes in oil and fatty acid contents under shade treatment. Bars represent the mean ± SE; * indicates significant differences (*p* < 0.05) between the shaded and control groups. ns indicates no significant difference (*p* < 0.05) between the shaded and control groups. (**D**) Interaction network between DEGs and DAMs in lipid biosynthesis. The blue circle represents genes; the red rhombus represents metabolites. The correlation coefficients were greater than |0.8|, with *p* < 0.05. The gray solid and dotted lines indicate positive and negative correlations, respectively.

As shown in Figure 7A (Table S4), the genes involved in lipid synthesis are significantly affected by shade treatment. Biotin carboxylase (BC), biotin carboxyl carrier protein (BCCP), and carboxyl transferase subunit alpha (CTα) are three subunits of Acetyl-CoA carboxylase (ACCase) [34,35,36]. Eight *ACCase* genes were slightly upregulated at 60 DAP, and seven *ACCase* genes were slightly downregulated at 90 and 120 DAP in the shaded group compared to the control group. KASI, KASII, and KASIII are three types of enzymes of 3-oxoacyl-ACP synthase (KAS) that catalyze the synthesis of different carbon chains [37]. Compared to the control group, two *KASIII* genes were slightly upregulated at the early stage (60 DAP); however, with the increased treatment time, these genes were slightly downregulated at 90 and 120 DAP in the shaded group. One 3-ketoacyl-ACP reductase (*KAR*) gene was significantly downregulated at 60 DAP but significantly upregulated at 120 DAP in the shaded group compared to the control group. One 3-hydroxyacyl-ACP dehydratase (*HAD*) gene was slightly downregulated in all phases in the shaded group compared to the control group. Fatty acyl-ACP thioesterase A (FATA) and fatty acyl-ACP thioesterase B (FATB) had a significant impact on fatty acid content and carbon chain length [38]. FATA mainly hydrolyzes C18:1-ACP, while FATB mainly hydrolyzes C16:0-ACP and C18:0-ACP [39]. One *FATA* gene was slightly upregulated, while three *FATB* genes were slightly downregulated in all phases of the shaded group compared to the control group.

FAD exists in the endoplasmic reticulum and is the key enzyme for the formation of polyunsaturated fatty acids from monounsaturated fatty acids [40]. SAD is a crucial enzyme that catalyzes the conversion of 18:0-ACP to 18:1-ACP [41]. The expression levels of *SAD* and *FAD2* were universally higher at all stages, reaching their highest level at 90 DAP. The genes encoding *FAD2* and *SAD* were significantly downregulated at 90 DAP in the shaded group compared to the control group, with log2 FC values of −1.02 and −1.51, respectively. Six of seven *PDAT* genes and one *DGAT1* gene were slightly downregulated at 90 and 120 DAP in the shaded group compared to the control group. As can be observed in Figure 7B (Table S5), the metabolites involved in the lipid synthesis process were significantly affected by shade treatment. The downstream metabolite accumulation (LysoPC 18:1, LysoPC 18:2, LysoPC 18:3, and 66:21; TG (22:7/22:7/22:7)) and upstream gene expression were consistent. Compared to the control group, LysoPE 16:0, LysoPE 18:1, LysoPE 18:2, LysoPE 18:3, LysoPC 16:0, SQDG 32:8; SQDG (16:4/16:4), and Plasmenyl-PC 18:0; PC (P-14:0/4:0) were significantly decreased at maturity in the shaded group. The correlation analysis also showed that *FAD2* (LOC109001694) and *SAD* (LOC109012153) were positively correlated with Plasmenyl-PC 18:0; PC (P-14:0/4:0) (pos-M522T409) and LysoPC 18:1 (neg-M566T408) (Figure 7D, Table S6).

In addition, we performed an absolute quantitative analysis of fatty acids (Figure 7C, Table S8). In mature walnut embryos, the higher content of saturated fatty acids included palmitic (C16:0) and stearic (C18:0) acids, while the higher content of unsaturated fatty acids included oleic (C18:1), linoleic (C18:2), and linolenic (C18:3) acids [42]. At the mature stage (120 DAP), the contents of C16:0, C18:0, C18:1, C18:2, and C18:3 were significantly decreased by 22.26%, 29.73%, 59.90%, 7.23%, and 34.30%, respectively, in the shaded group compared to the control group. Meanwhile, the oil content levels in the shaded and control groups were 9.89% and 12.38% at 60 DAP, 52.73% and 60.87% at 90 DAP, and 61.28% and 69.14% at 120 DAP, respectively (Figure 7C, Table S7). The oil levels in the shaded group were slightly lower than in the control group at 60 DAP and significantly lower than in the control group at 90 and 120 DAP. We speculated that the decrease in fatty acid and oil content levels may have been caused by the downregulation of these genes involved in lipid synthesis, such as *FAD2* and *SAD*. In addition, one *HAD*, eight *LACS*, three *FATB*, two *PAP*, two *LPCAT*, and three *LPAAT* genes were slightly downregulated in all phases for the shaded group compared to the control group, which may have also led to the reduction in fatty acid and oil content levels. However, some genes presented contrasting expression patterns, indicating the complex biosynthetic mechanism of walnut oil under shade-treatment conditions. We also speculated that the downregulation of the gene expression hindered oil synthesis.

#### 2.6.2. Plant Hormone Signal Transduction in Response to Shade Treatment

**Figure 8:**
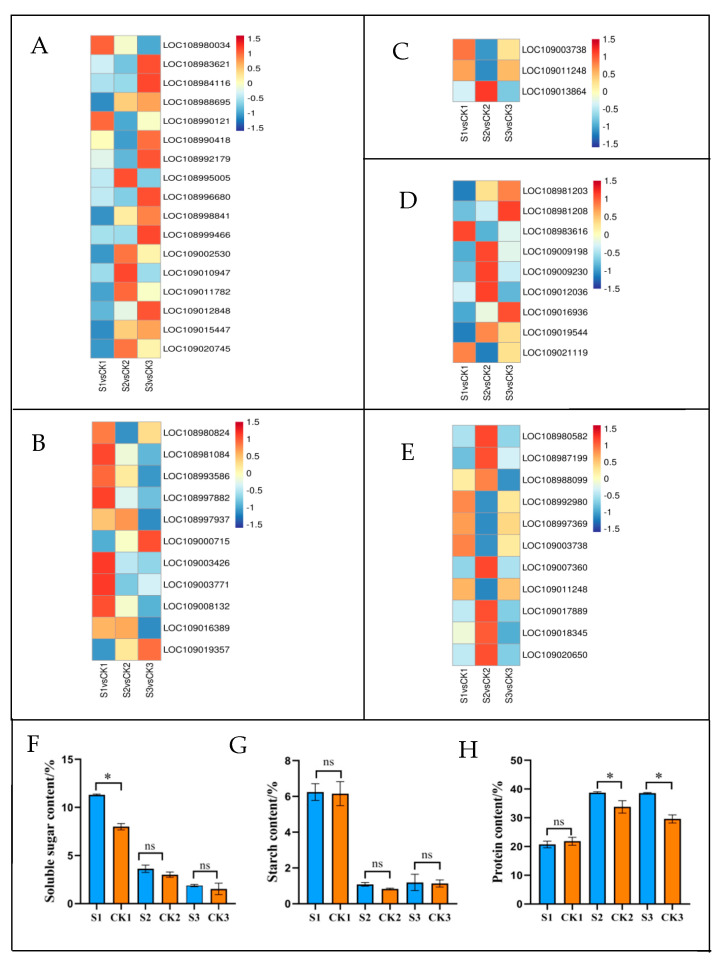
Transcriptional expression of DEGs in plant hormone signal transduction (**A**), starch and sucrose metabolism (**B**), glycolysis/gluconeogenesis (**C**), glutathione metabolism (**D**), and biosynthesis of amino acids (**E**) in shaded and control groups during different developmental periods. The log2 fold change in FPKM values was calculated under the shaded condition relative to the natural condition. Changes in soluble sugar content (**F**), starch content (**G**), and protein content (**H**) in walnut embryos under shaded treatment. Bars represent the mean ± SE; * indicates significant differences (*p* < 0.05) between the shade and control groups. ns indicates no significant difference (*p* < 0.05) between the shaded and control groups.

The plant hormone signal transduction mechanism was associated with 17 DEGs in this study (Figure 8A, Table S8). Gene encoding cyclin-D3-1 (LOC108990418) was significantly downregulated at 90 DAP in the shaded group compared to the control group. The genes encoding the histidine-containing phosphotransfer protein (LOC108995005, LOC109010947, and LOC109011782) were upregulated at 90 DAP in the shaded group compared to the control group. The gene encoding auxin transporter (LOC108980034) was significantly downregulated at 120 DAP in the shaded group compared to the control group. The genes encoding xyloglucan endotransglucosylase/hydrolase protein 22 (LOC108983621) and putative indole-3-acetic acid-amido synthetase GH3.9 (LOC108984116) were upregulated at 120 DAP in the shaded group compared to the control group.

#### 2.6.3. Starch and Sucrose Metabolism Processes in Response to Shade Treatment

**Figure 9:**
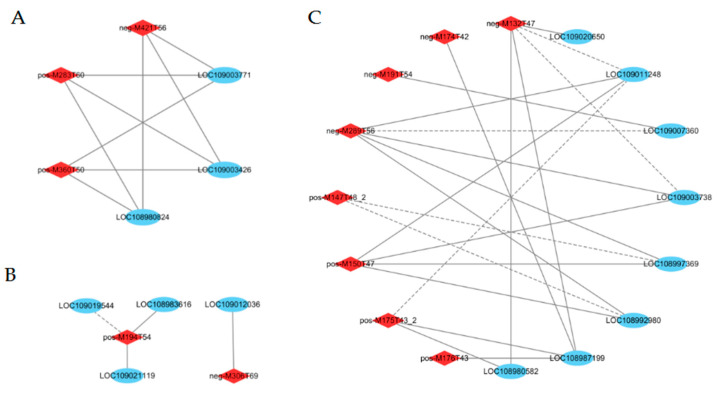
Interaction network between DEGs and DAMs in starch and sucrose metabolism (**A**), and glutathione metabolism (**B**), amino acid biosynthesis (**C**) processes. The blue circle represents genes; the red rhombus represents metabolites. The correlation coefficients were greater than |0.8|, with *p* < 0.05. The gray solid and dotted lines indicate positive and negative correlations, respectively.

Starch and sucrose metabolism was associated with 11 DEGs in this study (Figure 8B, Table S9). At 90 DAP, the genes encoding galacturonosyl transferase 8 (LOC108980824), sucrose-phosphate synthase 2 (LOC109003426), and glycogen phosphorylase (LOC109003771) were significantly downregulated in the shaded group compared to the control group. Starch and sucrose metabolism was associated with 3 DAMs in this study (Table 1 and Table S10). The levels of alpha, alpha’-Trehalose 6-phosphate, glucose 6-phosphate, and trehalose significantly decreased by 0.49-, 0.48- and 0.46-fold, respectively, at 90 DAP in the shaded group compared to the control group. To establish the association between genes and metabolites, we performed Pearson’s rank correlation tests. The correlation analysis we performed is presented in Figure 9A (Table S11). Glucose 6-phosphate (pos-M283T60), alpha, alpha’-Trehalose 6-phosphate (neg-M421T56), and trehalose (pos-M360T50) were positively correlated with galacturonosyltransferase 8 (LOC108980824), sucrose-phosphate synthase 2 (LOC109003426), and glycogen phosphorylase (LOC109003771). As shown in Figure 8F (Table S7), the soluble sugar content in the shaded group was significantly higher than in the control group at 60 DAP. However, with the increase in the treatment time, there were no significant differences in soluble sugar levels during middle and mature periods (90 and 120 DAP, respectively). As shown in Figure 8G (Table S7), there were no significant differences in starch content during all stages between the shaded and control groups. Shading treatment had little effect on the contents of soluble sugar and starch at the mature stage (120 DAP), which was consistent with the results obtained for the metabolomics.

#### 2.6.4. Glycolysis/Gluconeogenesis in Response to Shade Treatment

Glycolysis was associated with 3 DEGs presented in this study (Figure 8C, Table S12). The genes encoding ATP-dependent 6-phosphofructokinase 3 (LOC109003738) and phosphoglycerate kinase (LOC109011248) were significantly downregulated at 90 DAP in the shaded group compared to the control group. The gene encoding fructose-1,6-bisphosphatase (LOC109013864) was upregulated at 90 DAP in the shaded group compared to the control group.

#### 2.6.5. Glutathione Metabolism in Response to Shade Treatment

Glutathione metabolism was associated with 9 DEGs in this study (Figure 8D, Table S13). The gene encoding L-ascorbate peroxidase (LOC108981203) was significantly downregulated at 60 DAP in the shaded group compared to the control group. The gene encoding leucine aminopeptidase 1 (LOC108983616) was significantly downregulated at 90 DAP in the shaded group compared to the control group. The genes encoding glutathione peroxidase (LOC109009198, LOC109009230) and glutathione S-transferase (LOC109019544) were significantly upregulated at 90 DAP in the shaded group compared to the control group. The genes encoding glutathione S-transferase (LOC109016936 and LOC108981208) were significantly upregulated at 120 DAP in the shaded group compared to the control group. Glutathione metabolism was associated with 2 DAMs in this study (Table 1 and Table S14). At 90 and 120 DAP, the L-Glutathione level decreased significantly by 0.47- and 0.36-fold, respectively, in the shaded group compared to the control group. At 90 DAP, vitamin C significantly decreased by 0.47-fold in the shaded group compared to the control group. As shown in Figure 9B (Table S15), vitamin C (pos-M194T54) was positively correlated with glutathione S-transferase L3 (LOC109021119) and leucine aminopeptidase 1 (LOC108983616) levels. L-Glutathione (neg-M306T69) was positively correlated with glutathione S-transferase U8 (LOC109012036). These results show a complex regulatory mechanism occurring between the accumulation of metabolites in walnut embryos and the abundance of gene expression.

#### 2.6.6. Amino Acid Biosynthesis in Response to Shade Treatment

Amino acid biosynthesis was associated with 11 DEGs in this study (Figure 8E, Table S16). The genes encoding cysteine synthase (LOC108980582), anthranilate synthase beta subunit 2 (LOC108987199), glutamine synthetase (LOC108988099), arginase 1 (LOC109007360), and cystathionine beta-lyase (LOC109017889, LOC109018345, and LOC109020650) were significantly upregulated at 90 DAP in the shaded group compared to the control group. The genes encoding argininosuccinate synthase (LOC108992980), aminoacylase (LOC108997369), ATP-dependent 6-phosphofructokinase 3 (LOC109003738), and phosphoglycerate kinase (LOC109011248) were significantly downregulated at 90 DAP in the shaded group compared to the control group. The genes encoding glutamine synthetase (LOC108988099) were significantly downregulated at 120 DAP in the shaded group compared to the control group. Amino acid biosynthesis was associated with 13 DAMs in this study (Table 1 and Table S17). At 60 DAP, 2-oxoglutaric acid increased significantly by 2.04-fold in the shaded group compared to the control group. At 90 DAP, the levels of L-aspartic acid, L-citrulline, citric acid, glutamine, arginine, and citrulline were significantly increased by 9.28-, 2.65-, 2.15-, 2.04-, 3.24-, and 2.46-fold, respectively, in the shaded group compared to the control group. At 90 DAP, the levels of L-tyrosine, methionine, and D-ribose 5-phosphate decreased by 0.48-, 0.47- and 0.37-fold, respectively, in the shaded group compared to the control group. At 120 DAP, L-aspartic acid, L-citrulline, 2-isopropylmalic acid, pyruvic acid, and citrulline significantly increased by 2.22-, 8.92-, 3.19-, 4.60-, and 5.61-fold, respectively, in the shaded group compared to the control group.

Furthermore, DEGs and DAMs involved in amino acid biosynthesis presented strong correlations. As shown in Figure 9C (Table S18), L-Aspartic acid (neg-M132T47) was negatively correlated with phosphoglycerate kinase (LOC109011248) and ATP-dependent 6-phosphofructokinase 3 (LOC109003738) but positively correlated with cysteine synthase (LOC108980582), anthranilate synthase beta subunit 2 (LOC108987199), and cystathionine beta-lyase (LOC109020650). Arginine (pos-M175T43_2) was negatively correlated with phosphoglycerate kinase (LOC109011248) but positively correlated with cysteine synthase (LOC108980582) and anthranilate synthase beta subunit 2 (LOC108987199). Glutamine (pos-M147T48_2) was negatively correlated with aminoacylase (LOC108997369) and argininosuccinate synthase (LOC108992980). L-Citrulline (neg-M174T42) was positively correlated with anthranilate synthase beta subunit 2 (LOC108987199). In addition, the protein content increased from 18.51% in the control group to 24.12% in the shaded group during the mature period, which was significantly higher than in the control group (Figure 8H, Table S7). The high protein content can be attributed to the upregulated expression of synthetase genes after receiving shade treatment, which was consistent with the relative change that occurred in the total amino acid levels.

### 2.7. Analysis of Transcription Factors (TFs)

**Figure 10:**
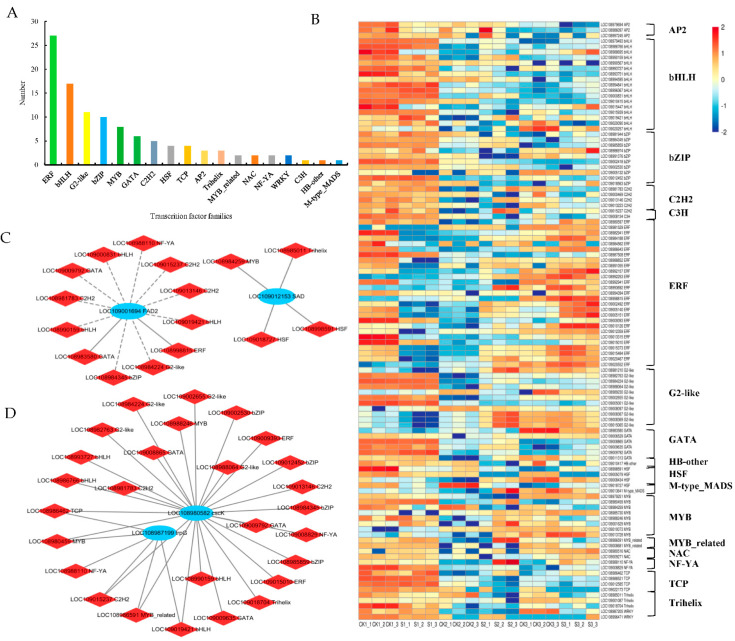
Analysis of differentially expressed transcription factors in walnut embryos under shade treatment. (**A**) Statistical analysis of transcription factor families. (**B**) Cluster analysis: red and blue blocks indicate the upregulation and downregulation of transcription factors, respectively. Connection network between DEGs (blue circle) and differentially expressed transcription factors (red rhombus) involved in lipid biosynthesis (**C**) and amino acid biosynthesis (**D**). The correlation coefficients were greater than |0.6|, with *p* < 0.05. The gray solid and dotted lines indicate positive and negative correlations, respectively.

A total of 109 differentially expressed TFs were detected under shade treatment (Figure 10A, Table S19), including ERF (27), bHLH (17), G2-like (11), bZIP (10), MYB (8), GATA (6), C2H2 (5), HSF (4), TCP (4), AP2 (3), Trihelix (3), MYB_related (2), NAC (2), NF-YA (2), WRKY (2), C3H(1), HB-other (1), and M-type_MADS (1). The expression of differential TFs is shown in Figure 10B (Table S20).

Pearson’s correlations were calculated between DEGs and differentially expressed TFs under shade-treatment conditions. TFs potentially associated with the lipid biosynthesis process in walnut embryos were also analyzed (Figure 10C, Table S21). The *SAD* gene showed a significant positive correlation with Trihelix (LOC108985011), MYB (LOC108984259), and HSF (LOC108998591 and LOC109018727). The *FAD2* gene was significantly positively correlated with GATA (LOC108983580) and ERF (LOC108998815) and significantly negatively correlated with bHLH (LOC108990159, LOC109000831, LOC109019421), bZIP (LOC108984345), C2H2 (LOC108981783, LOC109013146, LOC109015237), GATA (LOC109009792), and NF-YA (LOC108988110). TFs potentially associated with amino acid biosynthesis in walnut embryos were also analyzed (Figure 10D, Table S22). The results indicate that *trpG* is significantly positively correlated with NF-YA (LOC108988110) and bHLH (LOC109019421); *cscK* is significantly positively correlated with NF-YA (LOC109008829) and bHLH (LOC108990159, LOC108986766, LOC108993727, LOC109019421). These TFs may regulate genes involved in lipid and amino acid synthesis processes under shade treatment, which requires further validation in the research.

### 2.8. Weighted Gene Co-Expression Network Analysis (WGCNA)

**Figure 11:**
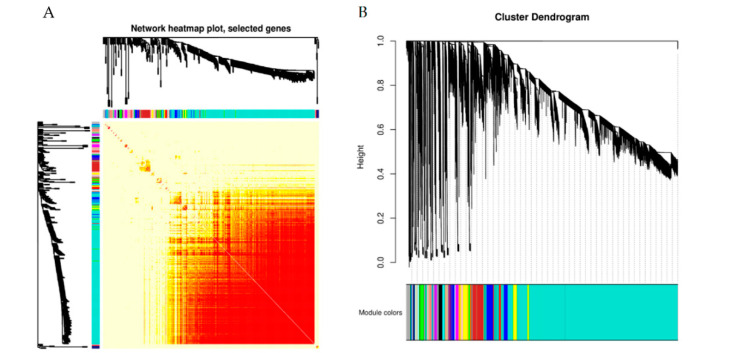

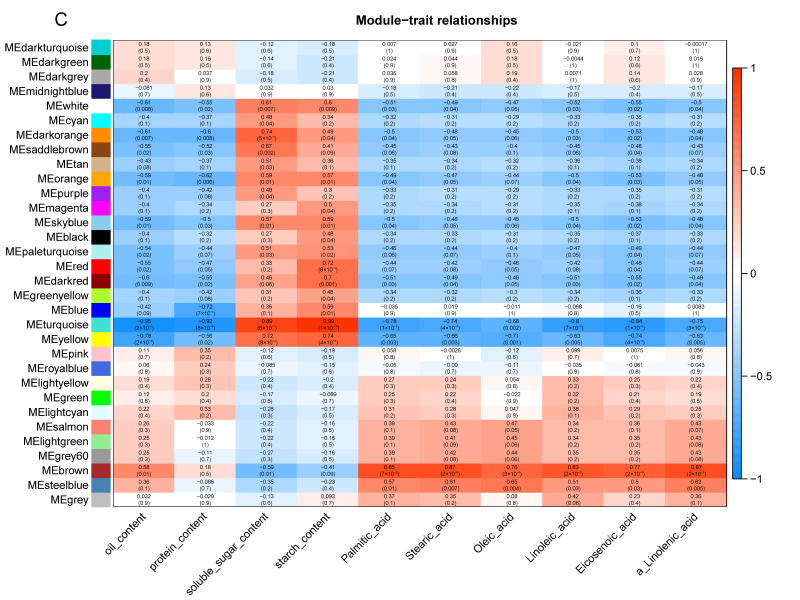
Co-expression of DEGs between control and shaded groups. (**A**) Network heat map plot. (**B**) Cluster dendrogram. (**C**) Module-trait relationships. The upper value in the block represents the correlation coefficient, and the lower value represents the *p*-value.

In our study, we concentrated on the expression patterns of genes to better understand the impact of shade treatment on the walnut fruit development process. The transcriptomic changes were investigated by WGCNA, which divided 30,993 genes into 32 modules (Figure 11A,B). The largest module (turquoise) contained 19,892 genes. More than 1000 genes were represented in blue-, brown-, yellow-, and gray-colored modules. As shown in Figure 11C, all the fatty acids and oils are positively correlated with the brown-colored module and negatively correlated with the turquoise-colored module. The soluble sugar and starch contents were positively correlated with the turquoise-colored module.

The genes in the brown-colored module presented the highest expression level at 120 DAP and were mainly enriched in thiamine metabolism (ko00730), plant hormone signal transduction (ko04075), tryptophan metabolism (ko00380), and glutathione metabolism (ko00480) activities (Figure S1A,C). The genes in the turquoise-colored module exhibited the highest expression level at 60 DAP and were mainly enriched in fatty acid metabolism (ko01212), protein processing in the endoplasmic reticulum (ko04141), fatty acid biosynthesis (ko00061), and the biosynthesis of amino acids (ko01230) (Figure S1B,D). Genes related to lipid synthesis were concentrated mainly in the turquoise-colored module. The gene co-expression networks in the brown- and turquoise-colored modules were constructed (Figure S1E,F). The results indicate that glycine-rich protein DOT1 (LOC109004676) and methionine-tRNA ligase (LOC108992747) are the core genes evident in the brown- and turquoise-colored modules, respectively. These two core genes may play an important regulatory role in fatty acid and amino acid synthesis.

### 2.9. Expression Validation of RNA-Seq Data by Real-Time PCR (qRT-PCR)

**Figure 12:**
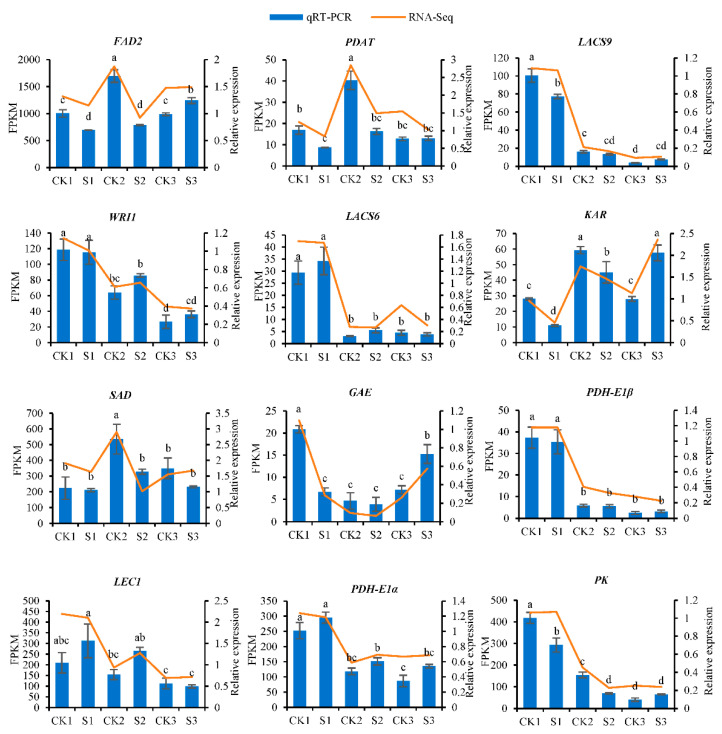
Expression patterns of 12 genes. The *y*-axis on the left represents the FPKM value obtained by RNA-seq; the *y*-axis on the right shows the relative gene expression levels analyzed by qRT-PCR. The *x*-axis indicates the different samples. Bars represent the average mean ± SE of three replicates. Different letters indicate significant differences at *p* < 0.05.

To verify the accuracy of the RNA-Seq data we obtained, we used qRT-PCR technology to validate 12 DEGs related to lipid biosynthesis and analyzed the expression patterns of these genes in walnut embryos obtained from the shaded and control groups. The results show that the expression patterns of these genes are consistent with the results obtained for the transcriptome sequencing we performed (Figure 12, Table S23).

## 3. Discussion

### 3.1. Oil Accumulation in Control and Shaded Groups

Walnut embryos contain rich nutrients (oil, protein, and fiber) and are also a favorite food item for human beings worldwide [43]. The growth and development processes of plants are closely related to the intensity of light they receive, which is an important environmental factor [44]. Under the constant variations exhibited in environmental factors, plants exhibit strong reactions and adaptability to ensure their growth and development [23]. Shade treatment lowers the oil content in walnut fruits [19]. Shade also affects the development of walnut trees [45]. This study systematically analyzed the effect of shade on the fruit-development process. The shade treatment we performed not only decreased the oil content but also increased the protein content in walnut embryos. This could be attributed to the weak light-intensity conditions affecting the growth and development of walnut trees, resulting in reduced photosynthetic property of walnut leaves and thus hindering the action of lipid biosynthesis. Interestingly, the content of soluble sugar in the shaded group was significantly higher than in the control group during the early stages of shade treatment, which may have occurred due to the effect of shade on the biosynthesis of walnut oil, resulting in a high level of sugar that could not be converted into oil. This result may have also been achieved due to a low-light stress condition, which increases the levels of soluble sugar present in the walnut embryo in order to resist fruit damage from the external environment.

### 3.2. Impact of Shade Treatment on Lipid Biosynthesis

The unsaturated fatty acid content and percentage values were determined by FAD [46]. SAD is the key enzyme of unsaturated fatty acid anabolism, which directly determines the total amount of unsaturated fatty acid present [47]. The *FAD2* gene, with a wide variety of putative response elements in its promoter, is responsive to multiple phytohormones and abiotic stresses; therefore, it can play an important role in stress-response behavior during plant growth and seed development stages [48]. Our study showed that the expression levels of *SAD* and *FAD2* genes were downregulated by shade, resulting in a decrease in C18:1, C18:2, and C18:3 levels. This result may have been achieved due to the destructive effect of a low-light stress environment on the metabolic system of walnut embryo development, resulting in a decrease in the material and energy supplied for walnut embryo development. Meanwhile, *FAD2* and *SAD* genes may be sensitive to low-light stress and regulated by complex signals; ultimately, the expression levels of these genes were inhibited and downregulated. Our research indicates that *FAD2* and *SAD* genes may play an important role in unsaturation fatty acid accumulation under low-light stress conditions.

Studies conducted on hickory embryos have shown that when the expression of *SAD* gene increased, the C18:1 content gradually increased [49]. We conducted a quantitative analysis of fatty acids and observed that shade treatment significantly reduced the content of C18:1. Previous studies have shown that the C18:1 content in soybeans decreased following exposure to shaded conditions [50]. The lower the C18:1 content, the lower the degree of oxidation and rancidity of the oil and the better the quality of the oil. Fatty acid composition and content were affected by growth environments [51,52]. Most of the genes (*PDAT*, *LACS*, *FATB*, *HAD*, *PAP*, *LPCAT*, and *LPAAT*) were slightly downregulated, which may result in a decreased level of oil content. We also observed that many precursors of lipid biosynthesis were significantly reduced in quantity under shade treatment. The downregulation of these genes may lead to a significant reduction in metabolites, fatty acid content, and oil content. However, the regulatory mechanism of shade treatment on the interaction between these genes and fatty acid components remains unclear in the literature. Further studies are required to elucidate this point.

### 3.3. Impact of Shade Treatment on Amino Acid Biosynthesis

Amino acids can provide a high level of nitrogen sources to plants. When plants are subjected to abiotic stress, they can resist harm by producing a high level of amino acids [53,54]. Shade can promote the accumulation of amino acids in both cotton [55] and tea leaves [56]. Our research indicates that the protein content in the shaded group is significantly higher than in the control group. Furthermore, the levels of L-aspartic acid, L-citrulline, and 2-isopropylmalic acid increased significantly under shade treatment. These metabolites may help to build physical and chemical barriers. Plants may protect themselves by synthesizing a large number of amino acids under abiotic stress conditions. Our research shows that the expression patterns of cysteine synthase (*cscK*) and anthranilate synthase beta subunit 2 (*trpG*) genes are upregulated under shade-treatment conditions, which is consistent with the accumulation patterns presented by most metabolites. This may be due to the fact that low-light stress conditions can induce the upregulation of amino acid synthesis genes, resulting in the production of a higher number of amino acids. When shade reduces the overall oil content level, the increased protein content may help plants resist fruit damage caused by low-light stress conditions.

### 3.4. Impact of Shade Treatment on TFs

TFs may potentially coordinate lipid biosynthesis activity occurring in walnut embryos in response to shade treatment. In our study, *NF-YA* (LOC108988110) and *bHLH* (LOC109019421) showed significantly different expression levels, which was upregulated in the shaded group compared to the control group. The expression levels of these TFs were opposite compared to the accumulation pattern of oil. HSF proteins play pivotal roles in the regulation of hormonal signal transduction and different abiotic stress responses [57]. *ERF* genes were highly responsive to the conditions of salt stress, drought stress, and ABA treatment [58]. The expression levels of *ERF* (LOC108998815) and *HSF* (LOC108998591) decreased in the shaded group, which may have regulated *FAD2* and *SAD* gene expression levels under shade-treatment conditions. Ultimately, we speculated that these transcription factors may have played an important regulatory role in low-light stress environments.

### 3.5. Integrated Analysis of Transcriptome and Metabolome Levels

**Figure 13:**
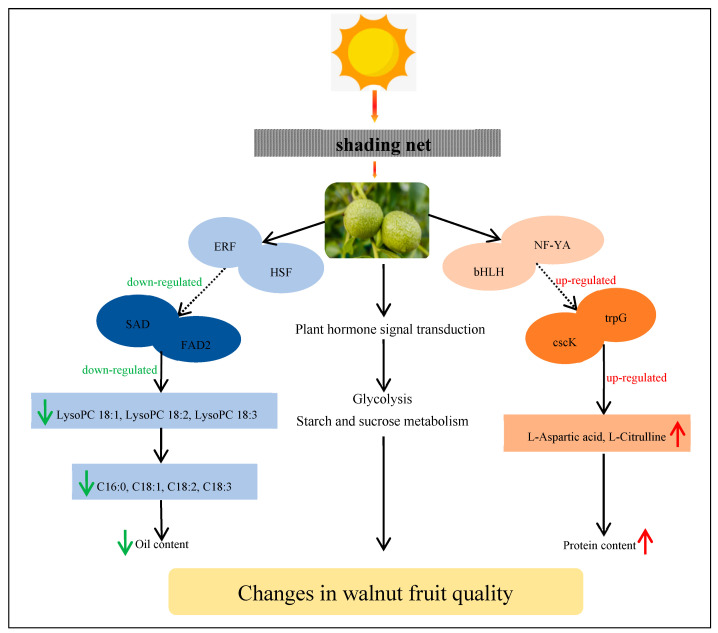
Model outlining the mechanism by which shade regulates the quality of the walnut fruit. This model uses *FAD2*, *SAD*, *cscK*, and *trpG* as key genes to positively regulate the biosynthesis of lipids and amino acids. In addition to lipid biosynthesis, other pathways are also regulated by shade treatment. This model also shows the shade mechanism regulating walnut fruit development. The red and green arrows indicate an increase and decrease, respectively.

The correlation analysis we conducted on metabolome and transcriptome levels showed that the expression of some genes in the walnut embryos were closely related to the content of metabolites exposed to shade treatment. It is possible that lipid biosynthesis in addition to glycolysis, starch, and sucrose metabolism and plant hormone signal transduction processes cooperatively interacted to modulate the oil content in the walnut embryos. Two key genes (*SAD* and *FAD2*) controlled the oil level in the walnut embryos by regulating the lipid biosynthesis levels (Figure 13). Additionally, we observed that most key genes were significantly downregulated at 90 DAP (middle stage of shade treatment), which may have been the period that was the most sensitive to the exposure to low-light stress conditions. We speculated that an appropriate light-intensity level was very important for successful walnut fruit development at this time.

## 4. Materials and Methods

### 4.1. Shade Treatment Used in a Walnut Plantation

The shade experiment was conducted at Hebei Lvling Fruit Industry Co., Ltd. (114°30′–114°33′ E, 37°29′–37°32′ N, Xingtai, China). Nineteen-year-old “Lvling” walnut cultivars were selected for the experiment. The setup of the shade-treatment experiment is presented in Figure 1A. Black nylon nets (Nongfeng Company, Taizhou, China) were placed 5 m above the ground, around 1 m over the walnut tree canopy. The shade experiment consisted of two groups: walnut trees with natural growth (control) and walnut trees exposed to 80–90% shading (S80–90%, transmitting 10–20% of natural sunlight). On 10 April 2020 (0 DAP), artificial pollination was performed on these trees. The nets were placed over the trees from 30 May (the embryos were only just visible, 50 DAP) to 10 August (the embryos were mature, 120 DAP) 2020. Walnut fruits exhibiting natural growth during the early (60 DAP), middle (90 DAP), and mature (120 DAP) stages of oil accumulation were labeled as CK1, CK2, and CK3, respectively. Walnut fruits exposed to shade were collected at 60, 90, and 120 DAP and were labeled S1, S2, and S3, respectively. The 60, 90, and 120 DAP results indicate time points at 10, 40, and 70 d, respectively, throughout the shade-treatment period.

A single replicate was obtained by 20 mixed fruits from three walnut trees. Three replicates were gathered to perform the transcriptome and physicochemical indices analysis, while six replicates were gathered for the untargeted metabolome analysis. Environmental factors were measured for the shaded and control groups to monitor the growth conditions of the walnut trees from the period 30 May to 10 August 2020, including the light-intensity, temperature, and humidity levels to which the walnut fruits were exposed (L99-LXWS). The sampled embryos were swiftly peeled prior to being rapidly frozen in liquid nitrogen and were moved to a freezer set at −80 °C for storage.

### 4.2. Determination of Physiological Indices during the Walnut Fruit-Development Process

The oil content was determined using the modified Soxhlet extraction method [59]. The fatty acid content was determined using gas chromatography [60], and the area of the chromatographic peak was used to perform the quantitative analysis. The contents of soluble sugar and starch were determined using the anthrone-sulfuric acid method [61]. The protein content was determined using the Kjeldahl technique [62].

### 4.3. Measurement of Photosynthetic Parameters

The photosynthetic parameters were measured in the experiment using the LI-6400 portable photosynthesis instrument (LI-COR Inc., Lincoln, NE, USA), from 6:00–18:00. We selected the top leaves of the mature compound leaves located on the periphery of the tree crown and then measured the net photosynthetic rate (Pn), transpiration rate (Tr), stomatal conductance (Gs), and intercellular CO_2_ concentration (Ci) values.

### 4.4. RNA Extraction and Library Construction

TRIzol reagent (Invitrogen, Carlsbad, CA, USA) was used to extract the total RNA, and NanoDrop ND-1000 (NanoDrop, Wilmington, DE, USA) was used to verify the purity of the RNA. SuperScript^TM^ II Reverse Transcriptase (Invitrogen, cat. 1896649, Carlsbad, CA, USA) was used to create cDNA. The samples were then sequenced at 2 × 150 bp paired-end runs on an Illumina Novaseq™ 6000 (LC-Bio Technology Co., Ltd., Hangzhou, China).

### 4.5. Bioinformatic Analysis of RNA-Seq Data and Co-Expression Network Construction

For the study, clean reads were obtained by removing adapter, ploy-N, and low-quality reads. FastQC was used to analyze the clean data we obtained [63]. HISAT2 [64] was used to map the data to the walnut genome (https://www.ncbi.nlm.nih.gov/assembly/GCA_001411555.2 (accessed on 6 January 2021)). The reads were then assembled using StringTie.

The differentially expressed mRNAs (DEGs) were based on |log2 fold change| ≥ 1 with a *p*-value < 0.05. We performed a KEGG pathway enrichment analysis (http://www.genome.jp/kegg/ (accessed on 8 January 2021)) on the genes. PlantTFDB (http://planttfdb.gao-lab.org/ (accessed on 8 January 2021)) was used to predict transcriptional factors [65]. The weighted gene co-expression network analysis (WGCNA) program was used to build the gene co-expression networks.

### 4.6. Quantitative Analysis

HiFiScript gDNA Removal RT MasterMix for qPCR (ComWin Biotech Co., Ltd., Beijing, China) was used to synthesize cDNA obtained from 1 μg of total RNA. MagicSYBR Mixture (ComWin Biotech Co., Ltd., Beijing, China) was used to perform the qPCRs. All primers are listed in Table S24. The relative gene expression level was calculated using the 2^−ΔΔCT^ method [66]. There were three biological replications.

### 4.7. Metabolite Extraction and LC-MS Analysis

The frozen walnut embryo was ground in liquid nitrogen. Approximately 100 mg of powder was weighed and mixed with 1 mL of 50% methanol. The mixture was then vortexed for 1 min, incubated for 10 min at room temperature, and stored overnight at a temperature of −20 °C. The mixture was then centrifuged at 4000× *g* for 20 min on the following day. Additionally, each mixture of 10 μL was mixed to produce pooled quality-control (QC) samples.

A TripleTOF 5600 Plus high-resolution tandem mass spectrometer (SCIEX, Warrington, UK) was used to analyze the samples. Chromatographic separation was performed using an ultraperformance liquid chromatography system (SCIEX, UK). For the reverse-phase separation, we used an ACQUITY UPLC T3 column (100 mm × 2.1 mm, 1.8 µm; Waters, UK). Solvents A (water containing 0.1% formic acid) and B (acetonitrile containing 0.1% formic acid) composed the mobile phase. With a flow rate of 0.4 mL/min, the gradient elution conditions were as follows: 5% solvent B for 0–0.5 min, 5–100% solvent B for 0.5–7 min, 100% solvent B for 7–8 min, 100–5% solvent B for 8–8.1 min, and 5% solvent B for 8.1–10 min.

### 4.8. Metabolomics Data Processing

XCMS software was used to preprocess the obtained LC-MS data. Each ion was identified using comprehensive retention time and *m*/*z* data. The open-access databases KEGG and HMDB were used to annotate the metabolites by matching the exact molecular mass data (*m*/*z*) to those obtained from the database within a threshold of 10 ppm. We used MetaX to preprocess the peak-intensity data we obtained [67]. The features that were detected in less than 50% of the QC samples or 80% of the biological samples were removed; the remaining peaks with missing values were imputed with the k-nearest neighbor algorithm and normalized using the probabilistic quotient normalization method. In addition, the relative standard deviations of the metabolic features were calculated across all QC samples, and those with standard deviations >50% were removed.

The group datasets were normalized prior to the analysis being performed. The data normalization step was performed on all samples using the probabilistic quotient normalization algorithm. Then, a QC-robust spline batch correction was performed using the QC samples. The *p*-value was analyzed by Student’s *t*-test, which was then adjusted for multiple tests using the FDR (Benjamini–Hochberg) for the selection of differential metabolites. The differential metabolites were screened with ratios ≥ 2 or ≤1/2, q value ≤ 0.05, and VIP ≥ 1.

### 4.9. Statistical Analyses

Genes and metabolites were analyzed using the OmicStudio tool (https://www.omicstudio.cn/tool, accessed on 8 January 2021). Significant differences were determined by ANOVA and Duncan’s multiple range test (*p* < 0.05). Excel 2010 (Microsoft, Redmond, Washington, DC, USA) was applied to draw the bar and broken-line graphs. Cytoscape (v3.7.1) was used to create the correlation network.

## 5. Conclusions

The data we collected in this study reveal the effects of shade treatment on lipid biosynthesis activity that occurs in walnut embryos. In comparison to the control group, the protein level increased significantly, whereas the oil content decreased significantly in the shaded group at the maturity level. Shade treatment also regulated lipid and amino acid synthesis, plant hormone signal transduction, and glycolysis pathway processes. Transcriptomics and metabolomics analyses revealed that a large number of DEGs and DAMs were filtered in the shaded groups. The expression patterns of *FAD2* and *SAD* genes were consistent with the accumulation of the majority of metabolites involved in lipid biosynthesis. The levels of fatty acids, such as C18:1, C18:2, and C18:3, decreased significantly in the shaded group. The results contribute to a better understanding of the research into the role of shade in overall walnut fruit quality.

## Figures and Tables

**Table 1:** Metabolites identified in three KEGG pathways during three stages of walnut fruit development in shaded and control groups.

KEGG Pathway	Compounds	60 DAP	90 DAP	120 DAP
		Ratio (S1/CK1)	Ratio (S2/CK2)	Ratio (S3/CK3)
Starch and sucrose metabolism (ko00500)	alpha, alpha’-Trehalose 6-phosphate	1	0.49	1
	Glucose 6-phosphate	1	0.48	1
	Trehalose	1	0.46	1
Biosynthesis of amino acids (ko01230)	L-Aspartic acid	1	9.28	2.22
	2-Oxoglutaric acid	2.04	1	1
	L-Citrulline	1	2.65	8.92
	2-Isopropylmalic acid	1	1	3.19
	L-Tyrosine	1	0.48	1
	Citric acid	1	2.15	1
	D-Ribose 5-phosphate	1	0.37	1
	Pyruvic acid	1	1	4.6
	Glutamine	1	2.04	1
	Methionine	1	0.47	1
	Arginine	1	3.24	1
	Citrulline	1	2.46	5.61
	(-)-Riboflavin	1	1	0.49
Glutathione metabolism (ko00480)	L-Glutathione	1	0.47	0.36
	Vitamin C	1	0.47	1

Note: The red blocks indicate upregulated metabolites; the blue blocks indicate downregulated metabolites; the yellow blocks indicate non differential metabolites.

## Data Availability

The transcriptome sequencing raw data were deposited in the National Center for Biotechnology Information Sequence Read Archive (NCBI SRA) under the Bioproject ID PRJNA983058.

## Supplementary Materials

The following supporting information can be downloaded at: https://www.mdpi.com/article/10.3390/ijms241310871/s1.
